# Supplementary material for: Family planning self-care: From global frameworks to local meaning, perceptions, experiences and opportunities in Niger
Source: PLOS Glob Public Health. 2026 Aug 31;6(8):e0006341. doi: 10.1371/journal.pgph.0006341 (PMC13529021; doi:10.1371/journal.pgph.0006341)
Supplement: Supplementary file 1 [file pgph.0006341.s001.docx]

Inclusivity in global research

PLOS’ policy on inclusivity in global research aims to improve transparency in the reporting of research performed outside of researchers’ own country or community and ensures that PLOS publications reporting global research adhere to high standards for research ethics and authorship. Authors of relevant research articles may be asked to complete the questionnaire below, which outlines ethical, cultural, and scientific considerations specific to inclusivity in global research. This questionnaire may be requested when researchers have travelled to a different country to conduct research, if research uses samples collected in another country, research with Indigenous populations or their lands, or if research is on cultural artefacts. Researchers travelling to another country solely to use laboratory equipment will not normally be required to complete the questionnaire. However, the questionnaire can be requested at the journal’s discretion for any submission – if you have been requested to complete this questionnaire by the PLOS journal you submitted to, please do so.

Please complete the questionnaire below and include this as a Supporting Information file with your manuscript. Note that if your paper is accepted for publication, this checklist will be published with your article in the supporting information files. Please ensure that you reference the checklist in the main body of your manuscript. We suggest adding a subsection ‘Inclusivity in global research’ to your Methods section and adding the following sentence: “Additional information regarding the ethical, cultural, and scientific considerations specific to inclusivity in global research is included in the Supporting Information (SX Checklist)”

The questions have been designed to be applicable to a wide range of study types, and there are subsections for both human subjects research and non-human subjects research. If any of the questions are not relevant to your research please mark them as “N/A” as appropriate.

**Ethical considerations, permits and authorship**

*This section is applicable to all research types.*

Provide details as to who granted permissions and/or consent for the study to take place in the Methods section of your manuscript. This should include the names of **all** ethics boards, governmental organizations, community leaders or other bodies that provided approval for the study. If individuals provided approval refer to these people by their role or title but do not list their name(s).

Reported on page number: 6

If there were any deviations from the study protocol after approval was obtained please provide details of these changes in the Methods section of your manuscript.

Reported on page number: NA

Did this study involve local collaborators that are residents of the country where the research was conducted or members of the community studied? If you do not have any authors from said communities, please provide an explanation for this below.

Yes, this study involved local collaborators and includes an author from the country where the research was conducted. Our author byline includes Nouhou Abdoul Moumouni, who resides in Niger and is affiliated with our local partner organization, GRADE Africa, based in Niamey, Niger. GRADE Africa formally oversaw the data collection efforts in-country. Furthermore, to ensure the perspectives of the community were systematically integrated into the methodology, the research team purposively sampled and engaged 15 local community leaders from the urban Niamey and rural Zinder regions to participate in qualitative in-depth interviews. All listed authors meet the PLOS criteria for authorship.

Everyone listed as an author should meet PLOS’ criteria for authorship and all individuals who meet these criteria should be included in the author byline, rather than the acknowledgements. For further information please see the journal’s Authorship Policy.

Yes, I confirm that all listed authors meet the PLOS criteria for authorship, and all individuals who meet these criteria are included in the author byline.

**Human subjects research (e.g. health research, medical research, cross-cultural psychology)**

Did you obtain written informed consent from a representative of the local community or region before the research took place? How did you establish who speaks for the community? Details of written informed consent obtained from study participants should be reported separately in the Methods section of your manuscript.

Community entry and engagement were strategically facilitated and overseen by our local partner institution, GRADE Africa, which directed the data collection efforts within Niger. To establish who speaks for the community and ensure that local gatekeepers were systematically integrated into the research design, 15 community leaders from the selected urban (Niamey) and rural (Zinder) regions were purposively sampled and engaged through qualitative in-depth interviews. Furthermore, as explicitly detailed in the Ethics Statement within the Methods section of the manuscript, individual participation was strictly voluntary; individuals provided verbal consent for initial eligibility screening, and all eligible participants (including both survey respondents and qualitative interviewees) provided formal written informed consent prior to the commencement of data collection

How did members of the local community provide input on the aims of the research investigation, its methodology, and its anticipated outcome(s)?

Local community members and resident experts systematically shaped the research methodology and its execution through several mechanisms. First, the study design and data collection processes were directly overseen by our local institutional partner, GRADE Africa, ensuring that the operational methodologies were contextually grounded and aligned with local realities. Second, to guarantee cultural appropriateness and content validity, the quantitative survey instruments were rigorously pre-tested and validated in the dominant local languages (French, Haoussa, and Zerma) prior to full deployment. Furthermore, to ensure that community perspectives directly informed the qualitative dimensions of the study, 15 local community leaders from both urban (Niamey) and rural (Zinder) regions were purposively sampled and engaged as key informants during the in-depth interviews. Finally, the overarching study protocol and its scientific aims were formally reviewed and approved by Niger’s national ethical authority, the Comité National d’Ethique pour la Recherche en Santé (CNERS)

When engaging with the local community, how did you ensure that the informed consent documents and other materials could be understood by local stakeholders?

To guarantee full linguistic comprehension and cultural appropriateness among local stakeholders, all data collection materials, including the informed consent procedures and the quantitative survey instruments, were rigorously translated, pre-tested, and validated prior to deployment. Furthermore, all data collection was actively conducted in the dominant local languages of the specific study regions, namely French, Haoussa, and Zerma. By employing these culturally relevant languages and ensuring our instruments underwent prior contextual validation for content validity, we confirmed that both the ethical consent protocols and the technical concepts surrounding family planning were clearly and accurately understood by all participants

Will the findings of the research be made available in an understandable format to stakeholders in the community where the study was conducted (e.g. via a presentation, summary report, copies of publications, etc.)? Please provide details of how this will be achieved.

Yes. The findings of this study will be made available in an understandable format to local stakeholders and health authorities in Niger to inform context-specific family planning scale-up. Dissemination efforts will be actively coordinated through our local partner organization, GRADE Africa, ensuring that materials are linguistically and culturally accessible. Furthermore, we will leverage the broader Research for Scalable Solutions (R4S) project network—the consortium under which this study was conducted—which is specifically designed to translate research evidence into scalable, local programmatic guidance and summary briefs for community and health system stakeholders.

**Non-human subjects research using specimens/ animals collected as part of the study, or those housed in archival collections. Examples include archaeology, paleontology, botany and zoology.**

Did the permission you obtained from a local authority to perform the study include an agreement on access to outputs and benefit sharing? This may include procedures to enable fair distribution of the benefits and resources arising from the research performed. Please include any details of Prior Informed Consent and Benefit Sharing Agreements obtained. These may be required by field-specific regulations, for example the Convention on Biological Diversity (CBD) and the associated Nagoya Protocol.

NA

If the material used in your study was imported, please A) provide the year it was imported and B) indicate whether permits were obtained to import/export the materials used, C) provide details of any permits obtained. If this information is not available, please indicate this.

NA

If you used archival specimens, please state how the material used in your study was acquired by the institute it is held in and provide details of any permits obtained for the original excavations/ sample collection. If this information is not available, please indicate this.

NA

How was the potential cultural significance of the materials collected in your study to local communities considered in your research design? Were Indigenous peoples and/or local researchers and institutions involved with archaeological excavations / collection of specimens? If so, please provide a description of their involvement.

NA

If your manuscript includes photographs of human remains please indicate whether authors obtained permission from descendants or affiliated cultural communities to do so.

NA
